# Spatio-temporal epidemiology of the cholera outbreak in Papua New Guinea, 2009–2011

**DOI:** 10.1186/1471-2334-14-449

**Published:** 2014-08-20

**Authors:** Paul F Horwood, Stephan Karl, Ivo Mueller, Marinjho H Jonduo, Boris I Pavlin, Rosheila Dagina, Berry Ropa, Sibauk Bieb, Alexander Rosewell, Masahiro Umezaki, Peter M Siba, Andrew R Greenhill

**Affiliations:** Papua New Guinea Institute of Medical Research, Goroka, Papua New Guinea; Walter and Eliza Hall Institute, Melbourne, Australia; World Health Organization, Port Moresby, Papua New Guinea; Papua New Guinea National Department of Health, Port Moresby, Papua New Guinea; Department of Human Ecology, The University of Tokyo, Tokyo, Japan; School of Applied and Biomedical Sciences, Federation University, Churchill, Victoria Australia

**Keywords:** Cholera, *Vibrio cholerae*, Spatio-temporal distribution, Cluster analysis, Papua New Guinea

## Abstract

**Background:**

Cholera continues to be a devastating disease in many developing countries where inadequate safe water supply and poor sanitation facilitate spread. From July 2009 until late 2011 Papua New Guinea experienced the first outbreak of cholera recorded in the country, resulting in >15,500 cases and >500 deaths.

**Methods:**

Using the national cholera database, we analysed the spatio-temporal distribution and clustering of the Papua New Guinea cholera outbreak. The Kulldorff space-time permutation scan statistic, contained in the software package SatScan v9.2 was used to describe the first 8 weeks of the outbreak in Morobe Province before cholera cases spread throughout other regions of the country. Data were aggregated at the provincial level to describe the spread of the disease to other affected provinces.

**Results:**

Spatio-temporal and cluster analyses revealed that the outbreak was characterized by three distinct phases punctuated by explosive propagation of cases when the outbreak spread to a new region. The lack of road networks across most of Papua New Guinea is likely to have had a major influence on the slow spread of the disease during this outbreak.

**Conclusions:**

Identification of high risk areas and the likely mode of spread can guide government health authorities to formulate public health strategies to mitigate the spread of the disease through education campaigns, vaccination, increased surveillance in targeted areas and interventions to improve water, sanitation and hygiene.

**Electronic supplementary material:**

The online version of this article (doi:10.1186/1471-2334-14-449) contains supplementary material, which is available to authorized users.

## Background

Cholera was first reported in Papua New Guinea in July 2009 [1]. From July 2009 to late 2011 > 15,500 suspected cases of cholera and >500 deaths were reported throughout the country [2]. However, these figures are thought to be a substantial underestimate of the true morbidity and mortality associated with the outbreak [3].

Although more than 200 serogroups of *Vibrio cholerae* have been described only two serogroups, O1 and O139, are responsible for the epidemic cases of cholera that are reported throughout the world. The most important of these, the O1 serogroup, can be further divided into two biotypes, namely classical and El Tor, based on genetic and phenotypic differences [4]. In the last two decades there has been a rapid emergence of *V. cholerae* strains that contain a mixture of classical and El Tor genotypic and phenotypic characteristics, known as ‘atypical’ strains. Molecular analyses have demonstrated that the outbreak in Papua New Guinea was caused by an atypical El Tor strain, with a close relationship to strains that circulated in Southeast Asia from 1990–2004 [2].

In July 2009, cases of acute watery diarrhoea associated with severe dehydration and death were reported from the north coast region (Morobe province) of Papua New Guinea. Following an outbreak investigation by the National Department of Health and the World Health Organization (WHO), cholera was confirmed as the cause of the outbreak [1]. During the subsequent 2 years, cholera spread throughout most of the lowland regions of the country [2]. In order to better understand the spatial and temporal characteristics of the cholera outbreak in Papua New Guinea we employed geospatial analytical techniques using records from the national cholera database. Spatio-temporal mapping has been used previously to determine the epidemiological characteristics of cholera outbreaks; most notably (and in the first instance) by John Snow in 1855 [5], but also to describe recent epidemics [6, 7]. The aim of this study was to describe the spatio-temporal distribution of the cholera outbreak in Papua New Guinea and highlight potential mitigation strategies that could be used for future outbreaks of cholera.

## Methods

### The setting

Papua New Guinea is a tropical, developing country situated directly to the north of Australia on the eastern half of the island of New Guinea (the western half forms part of Indonesia). The country has a population of ~7 million and is characterized by a mountainous interior; and tropical rainforests, rivers and swamps throughout much of the lowland regions. The health indicators for Papua New Guinea are generally poor on a global scale, with metrics such as mortality in children <5 years of age (57/1,000 live births) and life expectancy (female: 63 years; male: 59 years) being among the lowest in the Western Pacific Region [8].

### Cholera data

The national cholera database was compiled though passive surveillance reports from provincial hospitals, cholera treatment centers, oral rehydration points and local health centres from throughout Papua New Guinea. In the absence of diagnostic capacity in most parts of the country, cholera cases were reported based on clinical presentation. Cases were recorded following the WHO standard case definition: “in an area where the disease is not known to be present, a patient aged 5 years or more develops severe dehydration or dies from acute watery diarrhoea; in an area where there is a cholera epidemic, a patient aged 5 years or more develops acute watery diarrhoea.” [9]. Case definitions were supplemented with laboratory data where possible. When suspected cases were reported in a previously unaffected province culture was conducted [1, 2]. Once a province had been culture confirmed as cholera positive children of any age with acute watery diarrhoea were included in the national database.

### Data analysis

Georeferenced residence locations for each individual case were not available; however, case data were distributed across 81 georeferenced health facilities. Data were aggregated as weekly case numbers per health facility. Since there is no reliable information on the population at risk, the Kulldorff space-time permutation scan statistic (in the software package SatScan v9.2) was used to identify clustering of cases in space and time [10]. This model makes minimal assumptions about the time, geographical location, or size of the outbreak, and it adjusts for natural purely spatial and purely temporal variation. Since preliminary analyses identified very large space-time clusters containing a number of statistically significant sub-clusters, the maximum spatial window for the analysis was set as a circle with a 100 km radius. This allowed for identification of subclusters and a better description of the local spread of the disease. In addition, we conducted a cluster analysis for only the first 2 months of the outbreak in Morobe province to better understand the likely routes of spread which lead to further dispersion across the country. Data were further aggregated at the provincial level to describe the spread of the disease in the affected provinces in Papua New Guinea.

### Ethical statement

This study was approved by the Papua New Guinea Institute of Medical Research Institutional Review Board and the Papua New Guinea Medical Research Advisory Council. No human-subject work was undertaken in the study and all data were anonymised before analysis.

## Results

During this outbreak >15,500 cases of cholera were reported through the national cholera surveillance system. Following cleaning and sorting of the database 12,411 entries were included for analysis. Entries were excluded where insufficient spatio-temporal data was recorded, or the location data could not be mapped to existing georeferenced databases.

Males were more likely to be recorded as cholera patients during the outbreak, constituting 54.0% of cases (deviation from expected frequency χ^2^ = 79.672, p <0.001). The median age of cholera cases, calculated from 11,236 patients with sufficient age information recorded, was 22 years (inter-quartile rage 5,35; mean age: 23); with an age range of 1 month to 90 years. Among the cases for whom age was recorded, the majority occurred in people aged 15–49 years (5,807, 51.7%). All other age groups were well represented with 24.1% of cases occurring in children <5 years (n = 2,707), 14.3% of cases occurring in older children aged 5–14 years (n = 1,603) and 10.0% of cases occurring in older adults aged >50 years (n = 1,119). No mortality data were available for analysis as mortality was reported as aggregated data.

**Figure 1:**
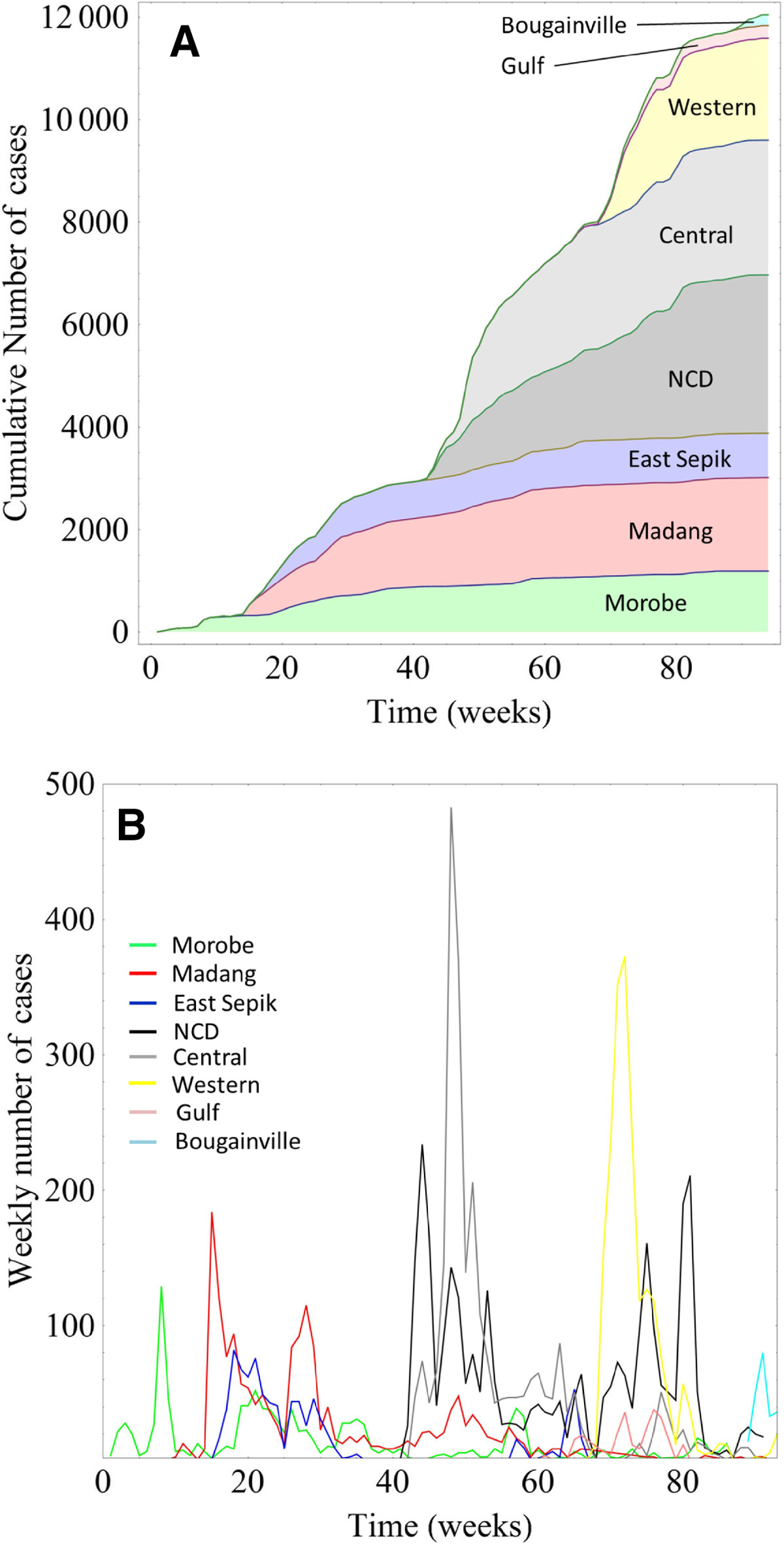
**Cumulative (Panel A) and weekly (Panel B) case numbers by province.** The distinct spikes in weekly case numbers indicate successive outbreaks in Morobe, Madang, East Sepik, National Capital District, Central District, Gulf, Western and Bougainville.

**Figure 2:**
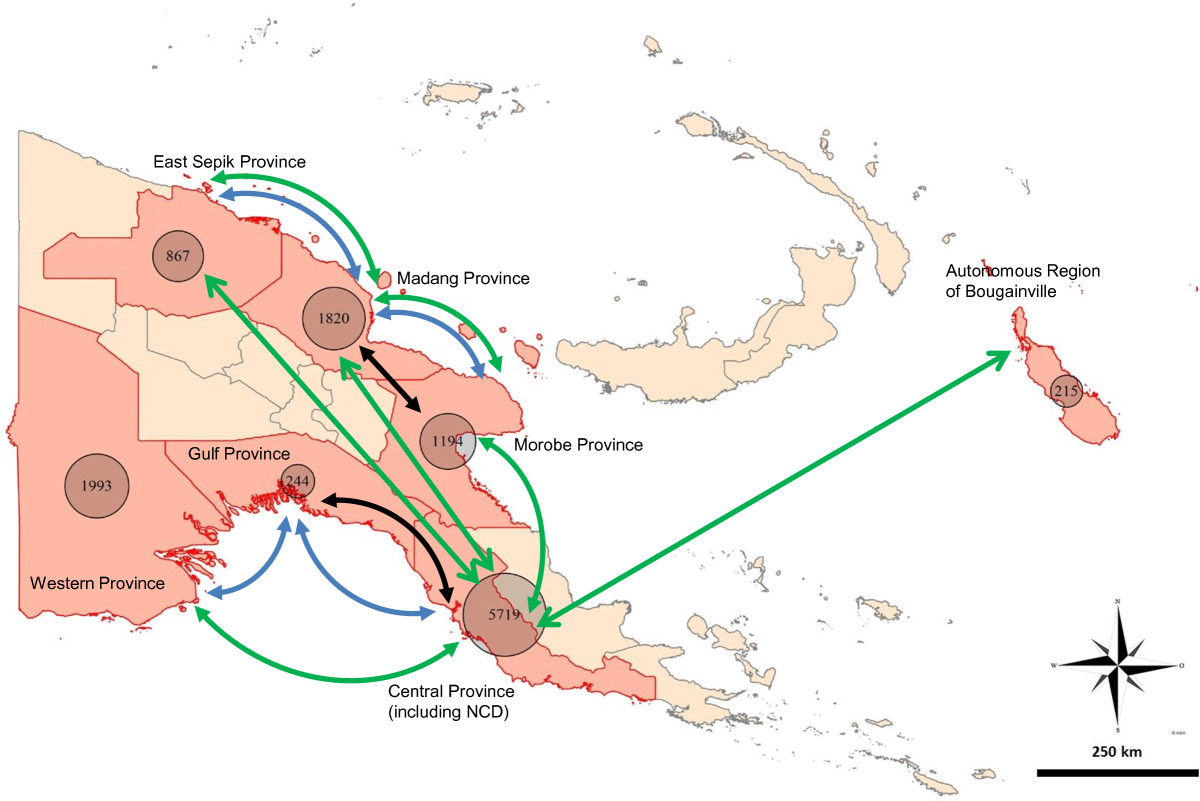
**Total case numbers and the main modes of transport between the affected provinces.** The figures are based on cases for which time and space data were available. The transport routes depict the main routes of transport available between provinces and do not represent the actual transport paths. Pedestrian transport is also important between neighbouring provinces, but is not depicted. The line colours indicate the different modes of transport: green - air travel; blue - boat; black - road. NCD - National Capital District.

The epidemic curve of the outbreak, for each affected province and the entire country, is presented in Figure 1. The provinces most affected by the outbreak were Morobe, Madang, East Sepik, National Capital District (NCD, which encompasses Port Moresby, the capital and largest city), Central, Gulf and Western; with the Autonomous Region of Bougainville also affected. The majority of the cases occurred in Central province and NCD. Figure 1 illustrates that there were distinct phases over the course of the outbreak, when the disease spread to new parts of the country; this would lead to an explosive propagation of cases, represented by either an increased slope in the cumulative number of cases (Figure 1A) or a spike in the weekly number of cases (Figure 1B). The geographical extent of the outbreak is summarized in Figure 2, showing the overall number of cases in each of the affected provinces (see Additional file 1 for an animation of outbreak clusters throughout the country). These numbers differ slightly from numbers previously reported by our research group [2]; however, we could not use all the data used previously for this analysis, as some cases were missing essential space-time data.

Additional file 1: An animation showing the progression of the cholera outbreak throughout Papua New Guinea. Circle sizes correspond to the weekly case numbers observed in each health facility (larger circles indicate more cases). The animation was constructed using Mathematica 9.0 (Wolfram Research Inc, Champaign, IL, USA). (MP4 573 KB)

**Figure 3:**
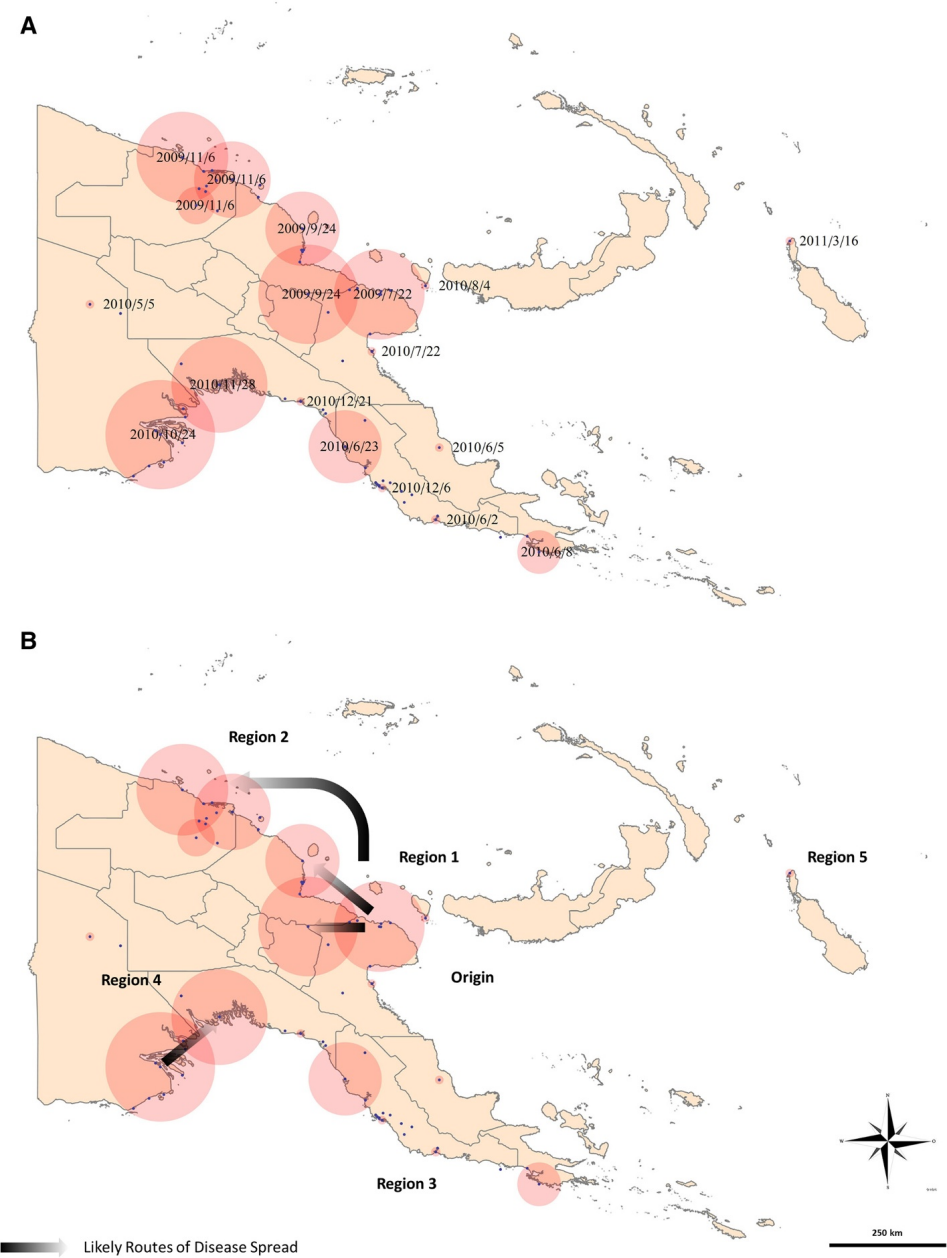
**Overall case clustering.** The outbreak can be grouped in 5 distinct spatio-temporal regions: Region 1: Morobe-Madang; Region 2: East Sepik; Region 3: NCD and Central province; Region 4: Western and Gulf provinces and Region 5: Autonomous Region of Bougainville. Panel **A**: Red circles indicate significant space-time clusters (radius to scale), the dates are the first day of each cluster. Panel **B**: Arrows indicate routes of likely disease spread.

**Figure 4:**
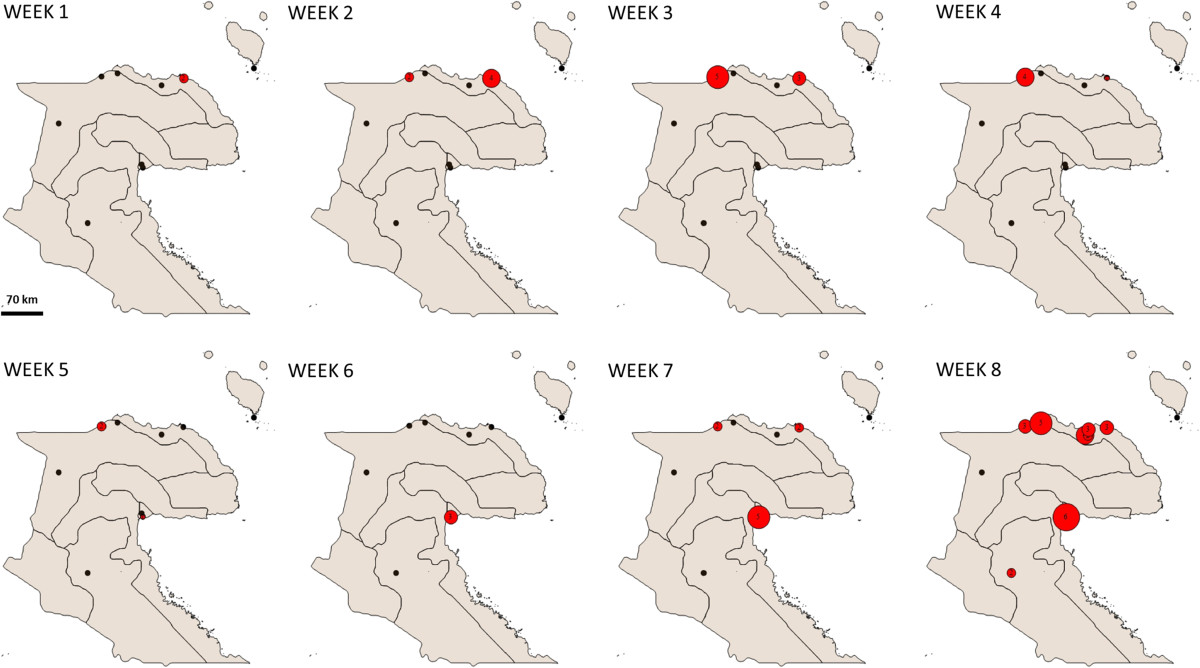
**Beginnings of the outbreak.** The first cases occurred on the North Coast of Morobe province. Starting from week 5 after the first reported case a steadily rising case number is observed in the capital city of Lae. This marks the start of the spread to other regions of PNG. Red circles indicated cases, black circles indicate health facilities. Numbers and circle sizes indicate cases per week in the specific locations.

**Figure 5:**
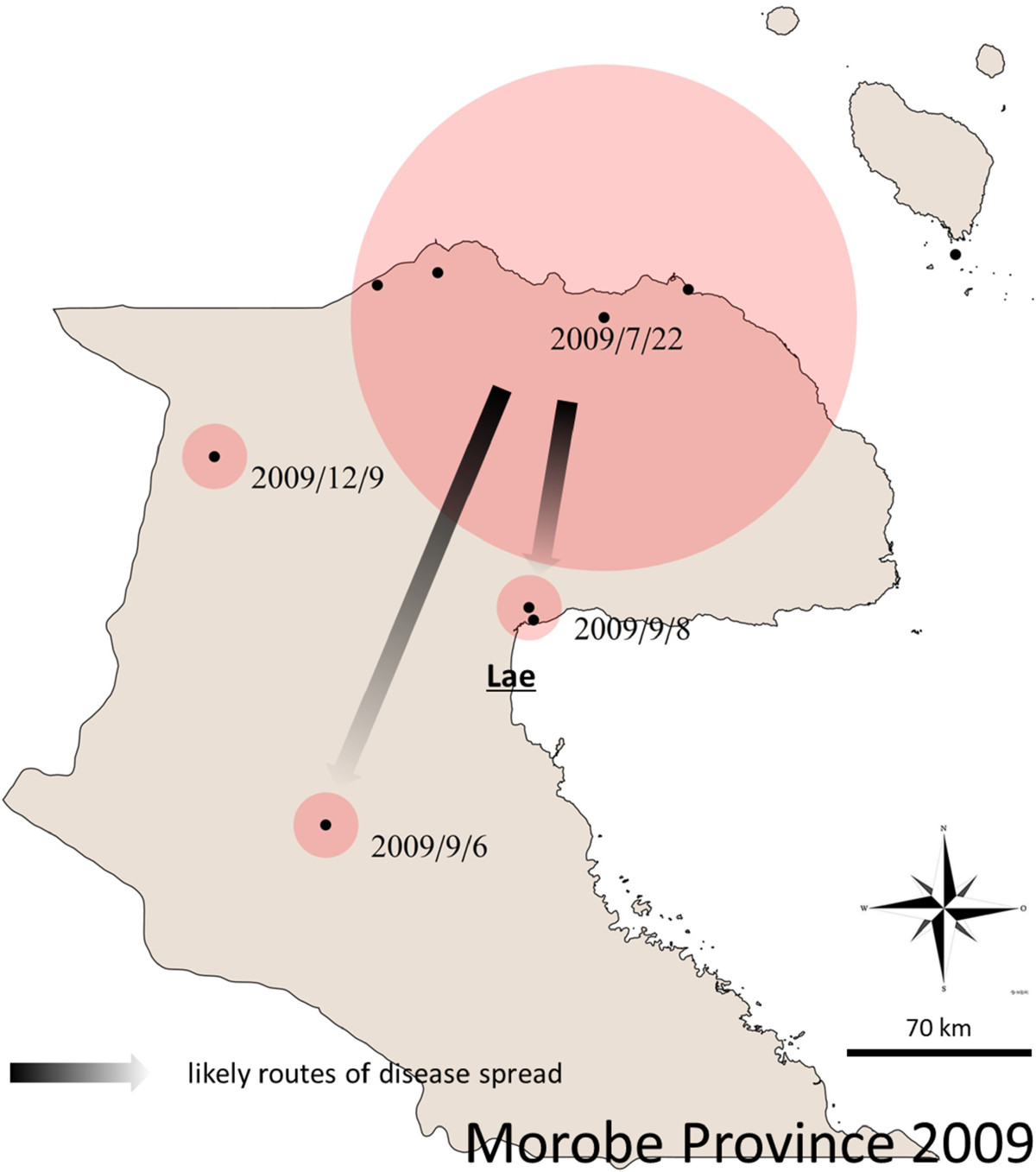
**Original Morobe space time clustering of cases.** When the Kulldorff Algorithm is applied to cases only in Morobe province and from Jul-Dec 2009, the original clustering on the Morobe North Coast is further resolved. The dates are the first days of each space-time cluster. Arrows indicate likely routes of disease spread.

The results of the spatio-temporal analysis (Figure 3A and 3B) further elucidate the dynamics of the disease spread, illustrating spread from the origin of the outbreak along the north coast of the country, and then to other coastal and island regions (see Additional file 2: Table S1 for details of the clusters). We paid particular attention to the first 8 weeks of the outbreak (Figures 4 and 5) as the early stages are the critical period to implement mitigation strategies to stop widespread transmission of the disease. The first cases were reported in health centres on the northern coast of Morobe province (22.07.2009), and in the first 4 weeks the outbreak was contained to this region. By the fifth week the outbreak reached the port city of Lae (Papua New Guinea’s second largest city), which serves as a transport hub for the region.

From Lae, the disease initially spread to neighbouring Madang province, most likely over land by road, with two significant space time clusters appearing to the west of the original cluster in late September 2009 including Madang town, the capital of Madang province.The disease reached East Sepik province by November 2009. However, by that time there were so many cases occurring on the north coast of Papua New Guinea that it is not possible to infer the exact route of this spread. Anecdotal reports suggest that pedestrian travel of people back to their clan area may have been an important mechanism of disease spread during the early stages of the outbreak. Madang and Morobe provinces are connected by road and shipping routes as well as frequent flights between the provincial capitals of Lae and Madang. East Sepik province is not connected to Madang and Morobe provinces by a road network, but frequent flights and regular shipping traffic connect Wewak (capital of East Sepik province) to the neighbouring provinces (Figure 2).

The main disease waves in these three provinces on the north coast had subsided by the end of 2009, although low case numbers persisted until late 2011. The disease was introduced to NCD in January 2010 and the surrounding Central province in May 2010 [2], with a peak in cases occurring from May to July 2010 in both provinces. NCD and Central were the most affected provinces with more than 5,700 cases for which we had spatio-temporal data (and over 7,000 cases in total: [2]). Port Moresby and the north coast are only connected by air travel (Figure 2).

**Figure 6:**
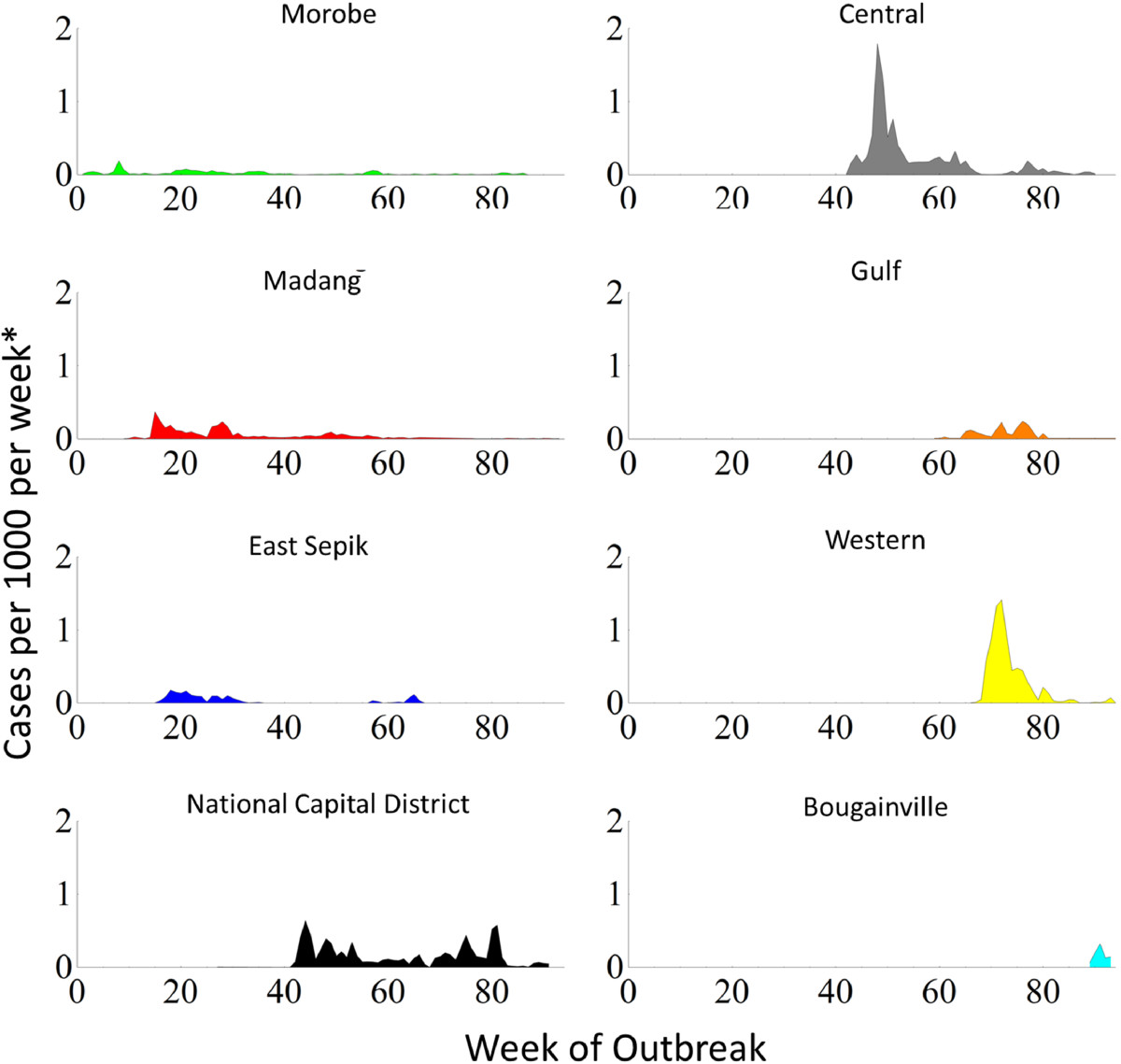
**Cholera incidence rate per week in each province.**

The outbreak in Port Moresby and Central province was succeeded by outbreaks in Western and Gulf provinces. Kerema, the capital of Gulf province, saw the first cases in August 2010 and Daru, the capital of Western province in October 2010, with the peak in cases for these provinces occurring in October and November, respectively. From Daru, the disease gradually spread northwest to other locations in Western province where there were frequently no health facilities and thus no line listed data to include for analysis.The last major outbreak occurred in the Autonomous Region of Bougainville. Again, case numbers increased rapidly. Bougainville is an island located at considerable distance from the PNG mainland and is only connected to the rest of the country through air travel (Figure 2).The individual plotting of the incidence rate per week in the provinces (Figure 6) show that the outbreak profiles were not uniform between the eight provinces where local transmission occurred. In the provinces on the north coast (Morobe, Madang and East Sepik) the outbreaks were characterized by a relatively small number of cases per week but extended outbreaks of up to 2 years. In contrast, the outbreaks in Central and Western provinces were characterized by explosive outbreaks with no long-term local transmission. Although in Western province, this may be an inaccurate picture of the outbreak due to the challenges associated with reporting and monitoring in such a remote and inaccessible region. The outbreaks in Gulf province and the Autonomous Region of Bougainville were characterized by a small number of cases over a shorter duration than many of the larger outbreaks on mainland Papua New Guinea. The outbreak in NCD was unique as there was a sustained period of almost 1 year where large numbers of cases were continually reported each week. Due to the larger urban population and access to hospitals and clinics with improved capacity, reporting mechanisms are more advanced in this area.

## Discussion

In this study we have elucidated the spread of cholera throughout Papua New Guinea through spatio-temporal and epidemiological analyses. The disease transmission patterns in Papua New Guinea are greatly influenced by the lack of road networks throughout most of the country. Three distinct peaks in cholera transmission occurred (Figure 1B), which correspond to the outbreaks along the north coast (the first, broad peak), along the south coast and in the southwestern region of the country (with an additional small fourth peak corresponding to the outbreak in Bougainville). These regions are geographically separated from each other and not connected by road, river or pedestrian networks. Thus the most likely mode of spread of this epidemic to new regions of the country was through commercial air travel. Anecdotal reports also suggest that spread of cholera to new regions may have been facilitated by the custom of transporting (by plane) the deceased back to their home province. Local funerary practices of touching the body and preparation for burial may have contributed to the spread of the disease; and have been associated with cholera transmission in other settings [11–13]. Previous studies by our group confirmed that the strains circulating in the geographically separated regions of Papua New Guinea were of clonal origin and not the result of separate incursions of *V. cholerae*[2].

The initial outbreak commenced in July 2009 on the north coast of Morobe province and rapidly spread to the neighbouring Madang and East Sepik provinces. The rapid spread was likely facilitated by the road network connecting Lae and Madang; and the frequent air travel between Madang and Wewak (East Sepik province). The peak in cases on the north coast occurred from August to September 2009, but cases continued to be reported from these areas, at a much reduced level, for nearly 2 years after the initial outbreak, resulting in nearly 4,000 cases. In early 2010 the epidemic spread to the National Capital District and surrounding Central province on the south coast. More than 7,000 cases were reported from these areas, with a peak of infection occurring from May 2010 and continuing until early 2011. Both provinces reported cases until late 2011 and a further peak in cases was recorded in National Capital District towards the end of the epidemic. The other major outbreak was in Western province where the highest case fatality rate (8.8%) of the epidemic was recorded [2]. Nearly 4,000 cases of cholera were reported in Western province in a short period, from October 2010 until March 2011. Western province is one of the most isolated and inaccessible regions of the country, this hampered outbreak control efforts and may have also resulted in a considerable underestimate of the true extent of the outbreak in this area.

Fortunately, no transmission of cholera was reported from the heavily populated highland region of the country, where the majority of the population resides. Despite road networks connecting the highland region with Madang and Morobe provinces (and commercial flights linking the highlands with most coastal areas, including Port Moresby) only six imported cases from the lowlands were recorded throughout the epidemic. The lack of local transmission in the highland area is likely due to geographical factors such as the steep topography and the fast-flowing, non-saline rivers in this region which are not conducive to the spread and persistence of *V. cholerae*.

The median age of cholera cases associated with this outbreak was 22 years (mean age 23 years). This figure may be biased to a younger age group as many age entries were recorded as ‘adult’ (n = 377) and therefore could not be included in the analysis. Similarly, many age entries were recorded as ‘unknown’ (n = 774) as individuals in this population, particularly adults, do not know their age. Nevertheless, the mean age was comparable to outbreaks in Uganda (26 years) [14], Kenya (23 years) [15] and Indonesia (21 years) [16].

In addition to under-reporting of age, in this setting, further biasing patient mean age could be the common presentation of cholera-like, acute watery diarrhoea in young children infected with rotavirus or other non-cholera enteric pathogens. On account of this, the WHO standard case definition for cholera stipulates “…a patient aged 5 years or more…” [9]. However in this outbreak, once cholera was confirmed as present in a region, children under 5 years of age were included in the national cholera database. Although this increases the risk of the inclusion of children suffering non-cholera acute watery diarrhoea, a recent multi-country surveillance study (using laboratory confirmation) found that the incidence and mortality of cholera was greatest in children <5 years [17]. This suggests that the inclusion of children (under 5 years) in the national database was warranted in Papua New Guinea. The findings of Deen and colleagues, along with our data which shows that almost one-quarter of all cases were in children under 5 years old, highlights the need to have laboratory diagnosis conducted on children and adults in cholera outbreaks. Indeed, in the recent devastating outbreak of cholera in Haiti children <5 years were the most commonly reported age group for cholera cases (13.1%) [18].

A limitation of this study was the high number of cases (>3,000) in the database that were not included in the current analysis due to the absence of necessary data. During the cholera outbreak, already fragile health care systems were further stretched by the inundation of severe, life-threatening illnesses to understaffed and poorly equipped clinics throughout the country. Although this resulted in suboptimal completion of case report forms in some areas, we believe that the results outlined in this study and the conclusions reached are an accurate representation of the cholera outbreak in Papua New Guinea. Throughout the country the support for rural clinics by organisations such as the National Department of Health, WHO and Médecins Sans Frontières was vital for the treatment of patients and the accurate recording of line data.

Currently there is much conjecture about the mode of cholera spread to new geographical areas, with various theories of autochthonous emergence and human initiated spread being espoused [19–21]. The fact that we still do not have a clear understanding of the inter-epidemic spread of this important pathogen is disconcerting. Previously we have proposed that the spread of cholera into Papua New Guinea for the first time on record may have been a result of contaminated ship ballast water and ocean currents, as the site of the initial outbreak was a very isolated coastal village close to a major international shipping route [3]. Although it is unlikely that the exact mechanism of the pathogen’s spread into Papua New Guinea will be determined, it is imperative that further studies to understand the global, regional and local spread of cholera epidemics be conducted.

During the Papua New Guinea cholera outbreak the lack of road networks and the difficulties associated with traveling to different regions within the country was an important factor in limiting, or at least slowing, the spread of the disease. However, the benefits derived from this lack of transport network is likely outweighed by the difficulties in rapidly delivering medicines, supplies and personnel to outbreak sites in an attempt to mitigate outbreak spread and reduce mortality. In addition, the poor access to basic services (such as water, sanitation, medical services and electricity) and challenges in the maintenance of such services due to the lack of connectivity in the country may increase the potential for subsequent outbreaks.

## Conclusions

Our analysis of the cholera epidemic in Papua New Guinea highlighted the importance of rapid and aggressive mitigation efforts during the early stages of the outbreak, before multifocal spread overwhelms control efforts. Until the road networks are improved commercial air travel should be a focal point for the control of spread during future outbreaks of cholera and other readily transmissible infectious diseases. In particular, the custom of transporting deceased family members back to their home province for funerary practices could be forbidden when cholera is suspected as the cause of death; however, due to the sensitivities of breaking long-standing cultural practices, disinfection of patient bodies suspected of dying from cholera could be implemented, as this strategy was shown to be effective for limiting funeral-related outbreaks in Guinea-Bissau, West Africa [11].

Cholera cases are rare in countries where there is adequate access to safe water sources and sanitation. Although no cholera cases have been reported in the country since late 2011, the environmental and societal factors that promote the sustained spread of enteric diseases such as cholera remain [22]. It is clear that considerable investments need to be made in the provision and maintenance of services such as safe water and sanitation to prevent further outbreaks of cholera in Papua New Guinea.

## Electronic supplementary material

Additional file 2: Table S1: Additional details for the clusters shown in Figure 3. (ODS 16 KB)

Below are the links to the authors’ original submitted files for images.

Authors’ original file for figure 1

Authors’ original file for figure 2

Authors’ original file for figure 3

Authors’ original file for figure 4

Authors’ original file for figure 5

Authors’ original file for figure 6
