# Supplementary figures and images for: Spatio-temporal epidemiology of the cholera outbreak in Papua New Guinea, 2009–2011
Source: BMC Infect Dis. 2014 Aug 20;14:449. doi: 10.1186/1471-2334-14-449 (PMC4158135; doi:10.1186/1471-2334-14-449)

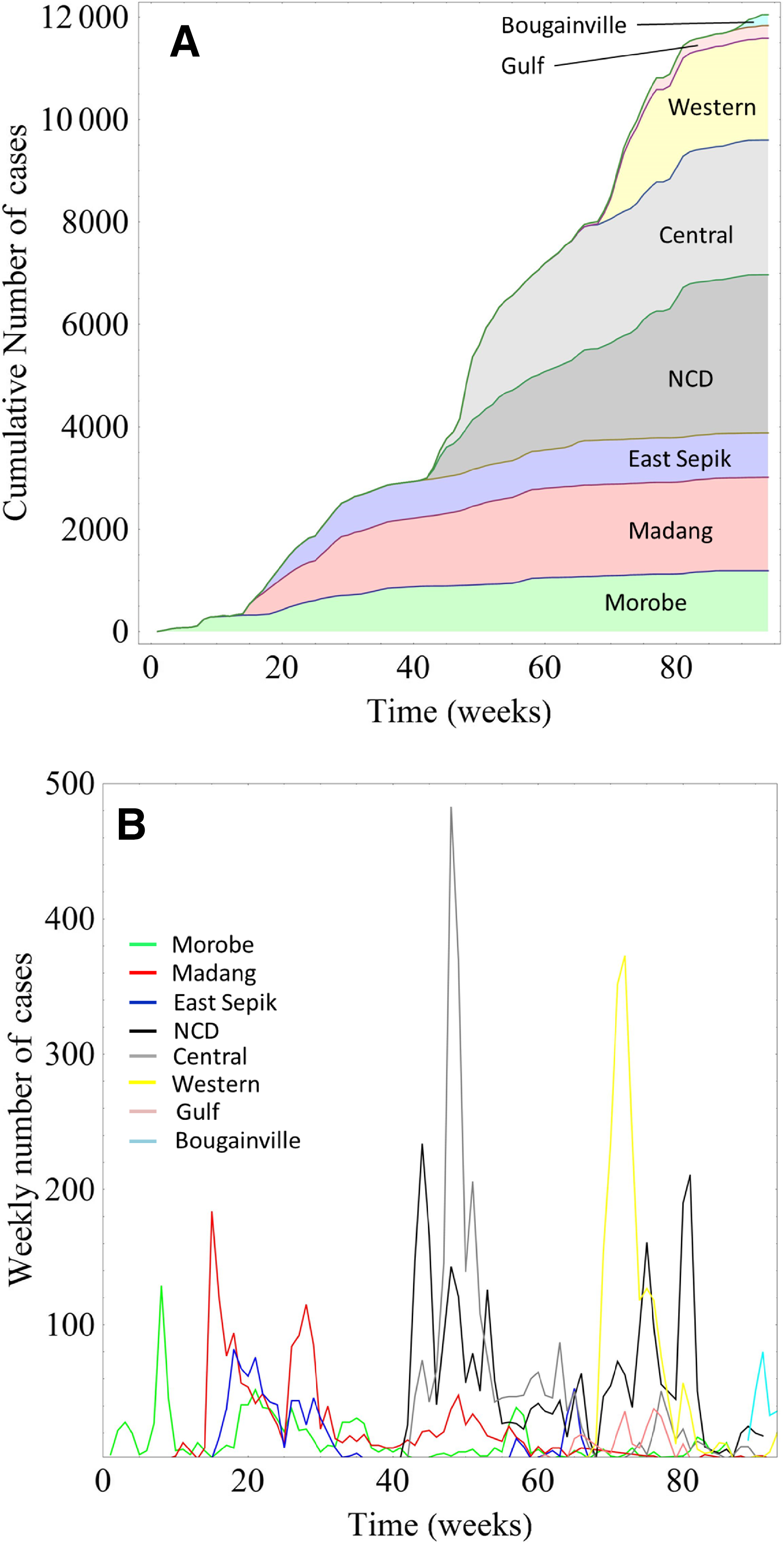

Supplement: Supplementary file 3 — Authors’ original file for figure 1 [file 12879_2014_3761_MOESM3_ESM.tif]

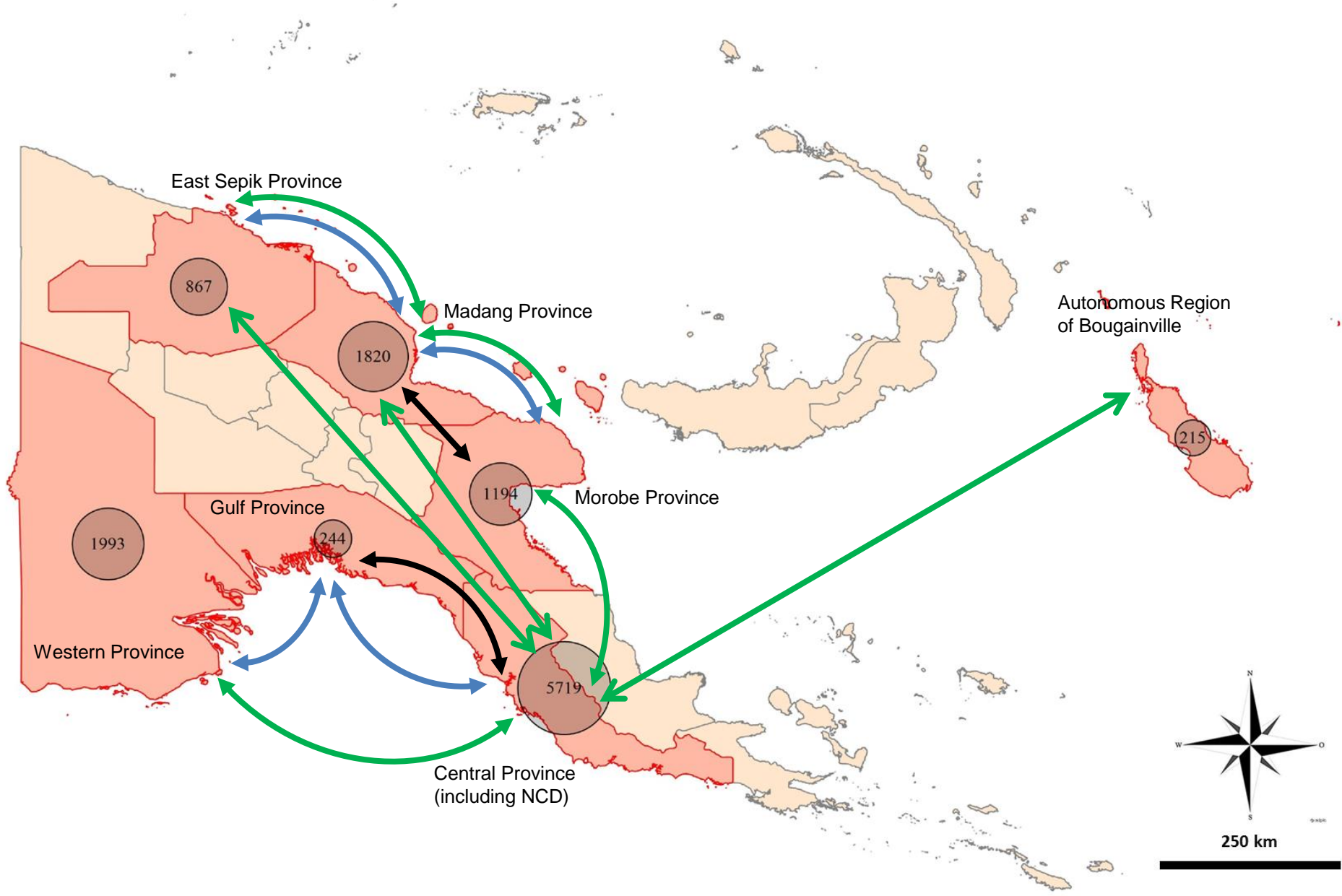

Supplement: Supplementary file 4 — Authors’ original file for figure 2 [file 12879_2014_3761_MOESM4_ESM.pdf]

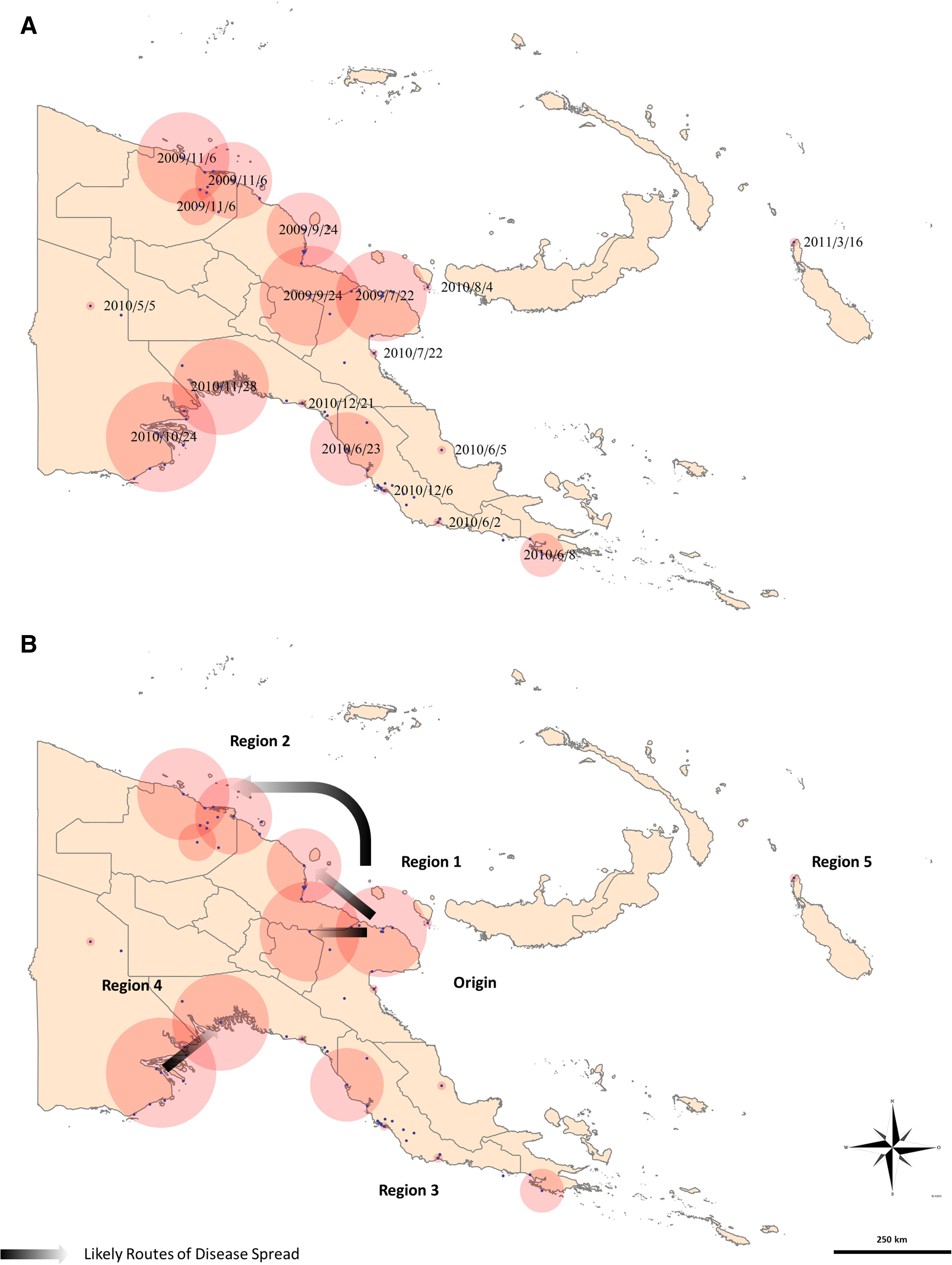

Supplement: Supplementary file 5 — Authors’ original file for figure 3 [file 12879_2014_3761_MOESM5_ESM.tif]

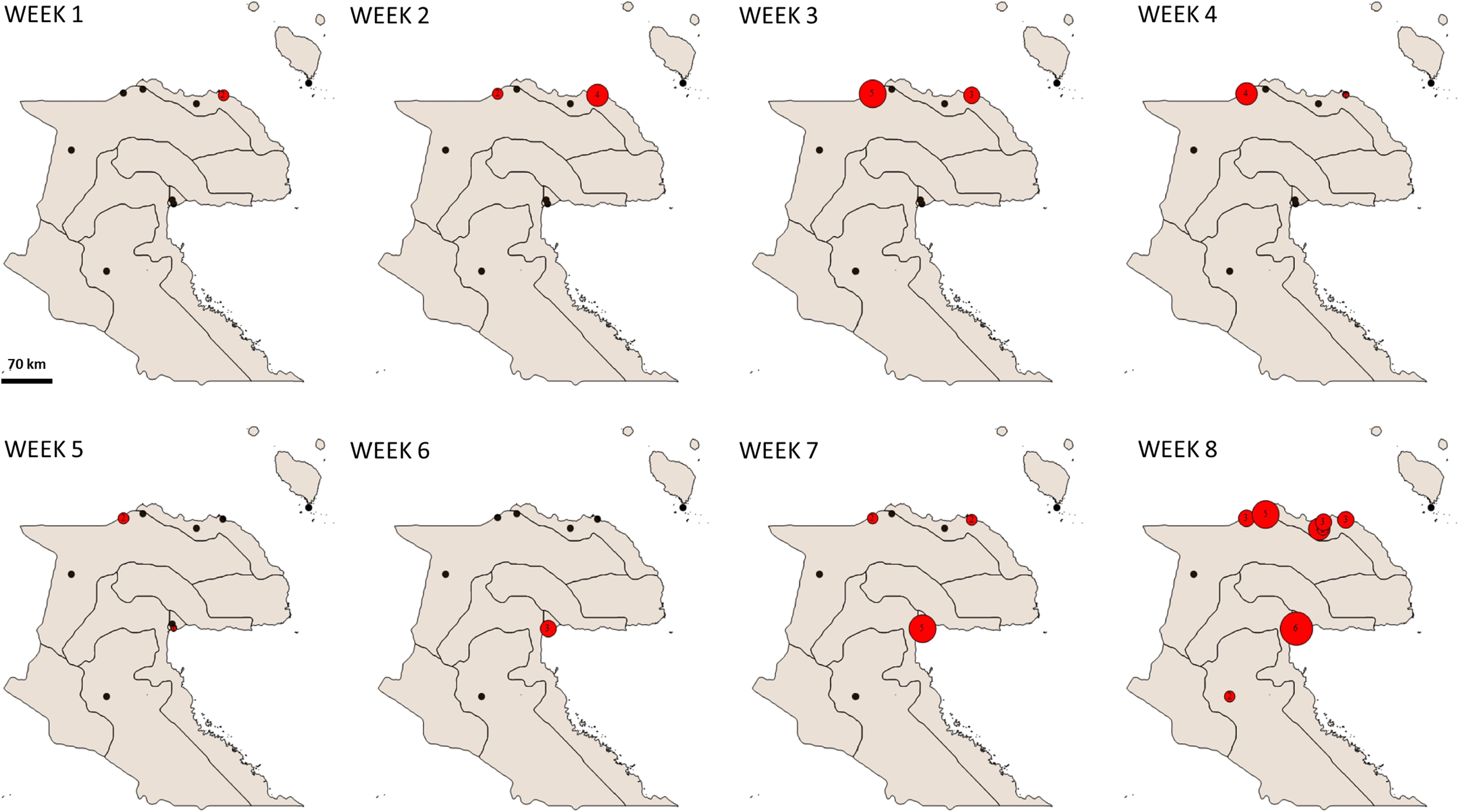

Supplement: Supplementary file 6 — Authors’ original file for figure 4 [file 12879_2014_3761_MOESM6_ESM.tif]

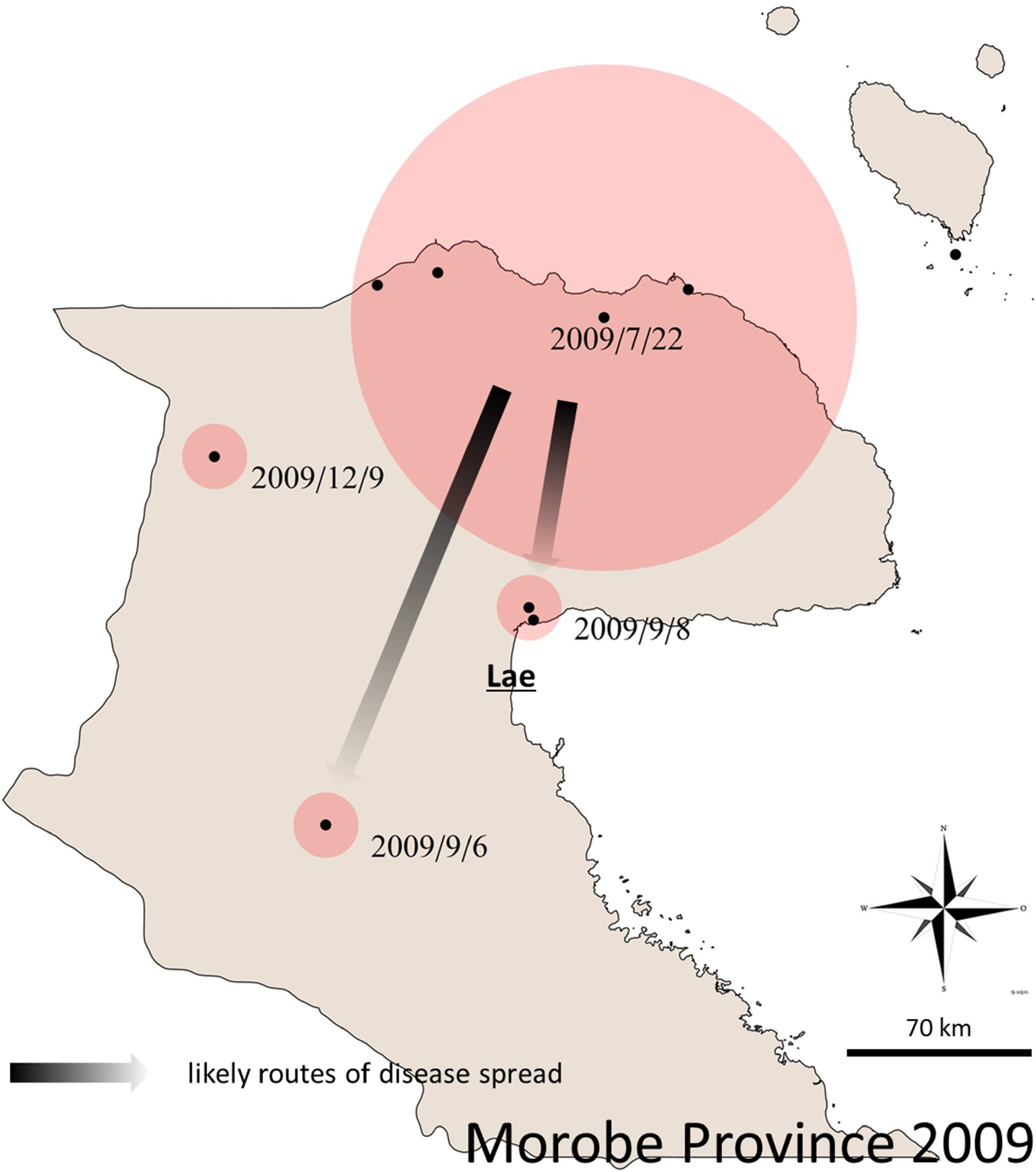

Supplement: Supplementary file 7 — Authors’ original file for figure 5 [file 12879_2014_3761_MOESM7_ESM.tif]

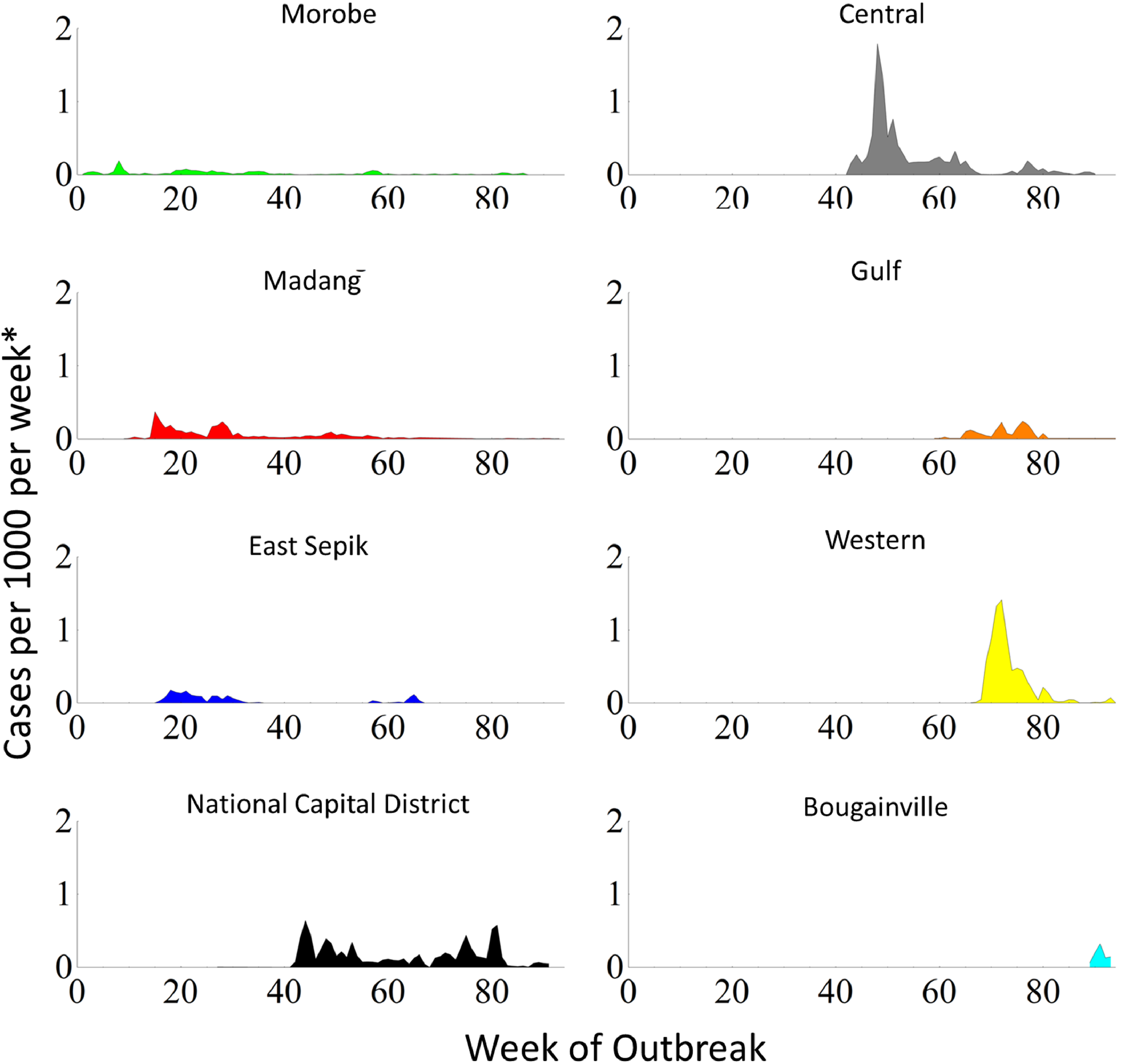

Supplement: Supplementary file 8 — Authors’ original file for figure 6 [file 12879_2014_3761_MOESM8_ESM.tif]
